# Mapping Adolescents’ Sense of Place and Perceptions of Change in an Urban–Rural Transition Area

**DOI:** 10.1007/s00267-019-01249-5

**Published:** 2020-01-14

**Authors:** Richard J. Hewitt, Florencia A. Pera, María García-Martín, Karl-Heinz Gaudry-Sada, Verónica Hernández-Jiménez, Claudia Bieling

**Affiliations:** 1 Informational and Computational Sciences Group, The James Hutton Institute, Craigiebuckler, Aberdeen, AB15 8QH Scotland UK; 2 Observatorio para una Cultura del Territorio (OCT), Calle del Duque de Fernán Núñez 2, 1, 28012 Madrid, Spain; 3 Department of Agricultural Economics and Rural Development, University of Göttingen, Platz der Göttinger Sieben 5, 37073 Göttingen, Germany; 4 Chair of Nature Conservation & Landscape Ecology, University of Freiburg, Tennenbacher Str. 4, 79106 Freiburg, Germany; 5 Instituto de Investigación Geológico y Energético, Av. de la República E7-263 y Diego de Almagro - Edificio Sky, Planta baja, 170518 Quito, Ecuador; 6 Institute for Social Sciences and Agriculture, Societal Transition and Agriculture (430b), University of Hohenheim, Schloss, 70599 Stuttgart, Germany

**Keywords:** Participatory mapping, Landscape perceptions, Landscape change, Urbanization, PPGIS

## Abstract

Landscapes are changing, with rural areas becoming increasingly urbanized. Children and adolescents are underrepresented in the sense-of-place literature. Our study aimed to understand how adolescent residents of a rural–urban transition area perceive and value their urbanizing landscape by examining sense of place and perceptions of landscape change. A Public Participation GIS approach, accompanied by a questionnaire survey, was applied to elicit responses from a sample of 747 students aged 12–18 in Colmenar Viejo, Madrid (Spain). Respondents’ sense of “self-in-place” or home range was small, around 1 km, although valued places were identified up to around 17 km away, and occasionally further afield. Most responses were associated with urban land, with clear difference between the urban core, strongly associated with emotions, and the suburbs, with activities. Functional locations (i.e. sports facilities) and places which were valued for their social meaning (i.e. shopping malls), could be differentiated. Students were perceptive about change processes in the urban area, but not about those on the peripheral semi-natural land. Younger children were less aware than older children of spaces outside of the town and carried out fewer activities there. Females carried out fewer outdoor activities than male adolescents. In contrast to the adult population, students were more strongly focused on urban areas than on their surrounding rural landscapes. Here, awareness-raising and incentives are needed, particularly those encouraging females into the use of areas beyond the urban land. Our results suggest a lack of meaningful integration between the core city and the periphery, with lessons for urban planners.

## Introduction

The world is increasingly urbanized. For the first time in human history, more than half of the global population lives in cities. This trend is prospected to continue and even accelerate (United Nations 2014). Peri-urban areas around existing urban nuclei are particularly dynamic, and typically see conversion of agricultural land to infrastructure and settlement areas and increasingly complex patterns of multifunctional land use (Simon 2008). Urbanization is considered one of the key global megatrends that modifies land use and consequently land cover, with profound implications for biodiversity and natural resources (Plieninger et al. 2016).

However, it would be inadequate to discuss urbanization only in terms of its biophysical effects for land cover. Perhaps even more important is the intangible socio-cultural dimension, where urbanization plays a role both as a driver for and as an expression of changes in people’s relationship to their environment. There is a vast literature, rooted in disciplines ranging from geography and psychology to economics and ethnography, that tries to conceptualize and evaluate human perception of and relation to nature and landscapes. This is pursued, for instance, in terms of sense of place (Jorgensen and Steadman 200; Perez-Ramirez et al. 2019), landscape values (García-Martín et al. 2018; Stephenson 2008), ecosystem services (Millennium Ecosystem Assessment 2003) or an *embodied perspective*, which emphasizes the role of experiences and activities such as recreational uses (Raymond et al. 2017).

Due to huge sustainability challenges posed within the era of the Anthropocene (Crutzen 2002; Steffen et al. 2011), understanding and mapping both the tangible and intangible dimension of changing landscapes has become a vibrant field of research (Hernández-Morcillo et al. 2017; Wu 2013). Approaches that link information on land cover, biodiversity or other aspects of landscape with data on human perception, valuation and activities are particularly relevant in this regard (Plieninger et al. 2015), Such approaches provide a means to link real and perceived change processes, which may not always align, something that has important implications for landscape planning and management (e.g., Bieling 2013).

One way of linking material and perceptual perspectives is through a public participation geographic information systems (PPGIS) approach, in which spatially explicit perception and evaluation statements are elicited from stakeholders and connected to existing maps of land cover or other natural science-based data (Brown and Fagerholm 2015). PPGIS studies typically involve participants creating or situating point, line, or polygon features in cartographic space to represent particular landscape attributes or elements, e.g., historic sites, areas of high nature value, or locations that are otherwise meaningful for them, also in the context of change processes (Brown and Weber 2012; García-Martín et al. 2017). Moreover, PPGIS is used to understand how specifically people locate and describe landscape values in space (Huck et al. 2014). PPGIS also allows for identifying areas of competing interests and resulting conflicts, for instance in the course of infrastructure development or in densely populated urban areas (Plieninger et al. 2018; Rall et al. 2017).

The majority of these studies has been undertaken with adult populations. Research into landscape perceptions of children and young people is longstanding, but less abundant. A selection of studies relevant to our present research is discussed as follows.

Owens (1988) interviewed 25 adolescents aged 14–18 from a predominantly white upper–middle-class town in California, USA about their landscape preferences. The author found notable differences in adolescent preferences when compared with adults, with these teenagers valuing most highly: natural spaces; places to be with their friends; places to be alone; places which from where they could see and not be seen; accessible places; and places they could call their own. Matthews et al. (1998), in a study of 13-year-olds from a socially and economically marginalized area of the Midlands, UK, found the group to be “active cultural producers” separate from adults through special places created by them and invested with their own values. The theme of adolescent distinctiveness, when compared with adults, is taken up by Mäkinen and Tyrväinen (2008) in their study of the uses of and values for green space of a sample of 300 adolescents aged 14–19 in Eastern Helsinki. In common with earlier research, the teenagers in this study valued green spaces highly and emphasized their importance as a meeting place. This was in contrast to adults sampled in a parallel study, which valued but did not meet in these areas (Mäkinen and Tyrväinen 2008). Abbott-Chapman and Robertson (2009) combined ethnographic and quantitative methods to explore the meanings of private and public space for a sample of young people aged 14–19 on the island state of Tasmania, Australia. These authors found gender disparity, with more girls than boys choosing familiar and home spaces, differences in age, with younger children preferring the town centre and older children friends’ homes or their local neighbourhood, and differences between city and country dwellers, with the former more frequently finding privacy in “my bedroom” and the latter finding privacy in nature. Sang et al. (2016) carried out a postal survey of residents living near green spaces in Gothenburg, Sweden, finding important variations by age and gender in the way green spaces were perceived and used. Older residents gave greater importance to green spaces for nature-related activities, even though the activities they carried out became less physically demanding as the respondents’ age increased. Yli-Panula et al. (2019) explored the landscape perceptions of Finnish and Swedish children aged 11–16 through their drawings, finding that children regarded both natural and built landscapes as worthy of preservation.

This brief review of the literature strongly supports the idea that children and teenagers perceive and relate to their environments in different ways than adults (for explorations into this see e.g. Alarasi et al. 2016; Travlou et al. 2008). Understanding young people’s perspectives is highly relevant given that it is their generation which will be the most strongly affected by the rapid, human-society driven transformation of landscapes and ecosystems. Moreover, this generation will also be in charge of future decision making and holds strong potential for civic engagement (Gordon et al. 2016) for instance in terms of landscape stewardship activities that may help steer human impact on nature in more sustainable directions.

In view of these considerations, the study we present here used a PPGIS approach to elicit information from a sample of adolescents about the way they perceive and value an urbanizing landscape. As a typical example of a rural–urban transition space we selected the Madrid metropolitan area in Spain, with a focus on the municipality of Colmenar Viejo (CV), a locality that has profoundly changed its character in the past decades, a process which is still ongoing. Conceptually, we anchor our study in the context of existing research into sense of place (Jorgensen and Steadman 2006). These authors, drawing in particular on environmental psychology literature, see sense of place as an umbrella term for a range of interrelated concepts around the idea of place. Our research design was thus targeted to draw out information relating to two such aspects: (1) place identity and; (2) place dependence (Jorgensen and Stedman 2006). Place identity refers to the set of feelings associated with particular physical settings (Proshansky et al. 1983) including how these settings provide meaning and purpose to life (Ujang and Zakariya 2015). This definition might also include a person’s sense of emotional attachment or connectedness to a particular place, sometimes treated separately as place attachment (Low and Altman 1992). Place dependence is defined by the connections between a physical setting and the activities that take place within it (Schreyer et al. 1981).

In our study, we sought to understand adolescents’ sense of place in their local landscape by eliciting data on their location choices for activities (e.g., meeting with friends, playing sport, walking in the country)—representing place dependence—and emotions (attachments to, and meanings provided by, particular landscapes, elements or aspects of landscape)—representing place identity.

Specific research questions were as follows:

1. Where, in the locality of CV, do respondents identify activities and emotions, and what does this tell us about their sense of place?
2. How do adolescents perceive landscape change processes, how does this match with change processes detected by analysis of land cover maps, and what preferences for future developments do they have?
3. How do the locations identified by the adolescents relate to land cover (biophysical landscape features) and what does this tell us about their relationship to their changing environment?
4. With what precision do adolescents spatially identify locations corresponding to activities, emotions and landscape change processes?

## Study Area

### Study Location and Background

**Fig. 1:**
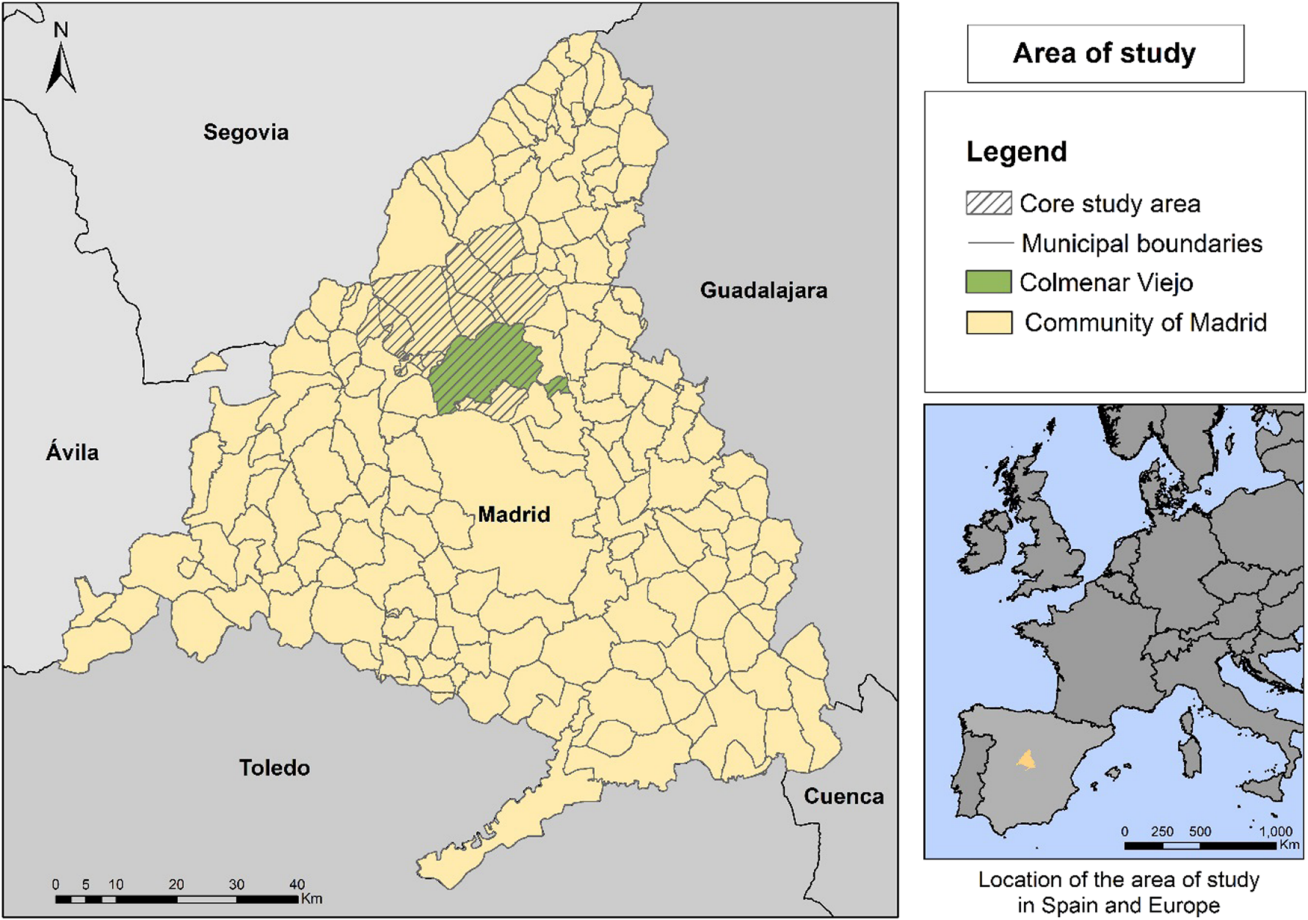
Study area, Colmenar Viejo, Madrid, Spain

CV is a municipality of the Community of Madrid with 49,498 inhabitants (INE 2019) and is located 30 km north of the city of Madrid (Fig. 1). It is situated approximately in the centre of the Castilian *meseta* (high plateau) in the foothills of the Sierra de Guadarrama.

The Madrid region has seen rapid and intensive growth of urban areas and infrastructure in recent years with important implications for sustainability and landscape values (López de Lucio 2003; Plata Rocha et al. 2009; Hewitt and Hernandez-Jimenez 2010; Hewitt and Escobar 2011; Gallardo and Martínez-Vega 2012; Díaz-Pacheco and Garcia-Palomares 2014). Although historically rural, with strong economic dependence on quarrying and livestock, the proximity of the capital city in an increasingly well-connected landscape of transport corridors, “big box” commercial centres, light industry and large-scale distribution hubs (de Santiago Rodríguez 2008; López de Lucio 2003) has transformed the study area in the last half century.

In recent years, CV and its immediate surroundings experienced significant land use change, in particular the expansion of housing, industry and infrastructures, mostly at the expense of agricultural land. The quarrying and working of granite, historically key to the town’s economy, has declined, whereas extensive pastoralism (cows, sheep and goats) still plays a central role in CV. A high proportion of land (30%) falls under natural protection as part of the *Cuenca Alta de Manzanares* Regional Park.

Demographically, CV concentrates most of its population in the age groups of 30–39 (17.6%) and 40–49 (17.1%). These groups are followed by the ages of 20–29 (12.8%) and 10–19 (11.3%). In comparison to other areas of the region, the municipality has maintained its demographic vitality with a total population growth of about 33% between 2001 and 2011. The entire Madrid region has grown in turn only 21% within the same period (INE 2019).

## Methods

### Data Collection

Data collection was carried out between July and October 2015 in groups of ~35 students across five secondary schools in CV municipality, facilitated by, and with the consent of, the relevant school directors and teachers. A total of 747 students between the ages of 12 and 18 participated in the study, covering the age group described by Piaget (1929) as “adolescence”, representing about 20% of the total population aged 12–18 of the municipality (estimates based on INE 2019). Both participants and their parents gave their informed consent for inclusion before they participated in the study. The study was conducted in accordance with the Declaration of Helsinki, a widely accepted worldwide standard intended to ensure ethical conduct in scientific research on human subjects.

To collect data from the participants, we used a web-based PPGIS approach (e.g., Fagerholm et al. 2016; García-Martín et al. 2017). Each participant was provided with a PC and an internet URL-address to an online questionnaire developed by the research team using the software Maptionnaire (https://maptionnaire.com/en/938) which they were asked to self-administer. Survey questions focused on landscape preferences, for which landscape-related activities and emotions were used as indicators (following García-Martín et al. 2017), as well as perceived landscape changes (Table 1).

**Table 1:** Survey questions and grouping according to landscape preferences and change perceptions, mapped to the specific aims of the research

Landscape preferences and change perceptions	Question
Activities	In this place I spend time outdoors in group. In this place I spend time outdoors alone. In this place I carry out some kind of sport outdoors. In this place I collect nature products.
Emotions	Here I perceive local history. Here I observed the environment’s ability to produce or renovate the air, soil and/or water. This place is emblematic for Colmenar Viejo. This place makes me think about the future. This place makes me think about the past. This place has major significance for me and my family. Here I perceive plants (flora), animals (fauna) or the ecosystem in general. Here I enjoy the beauty of the landscape in general.
Changes	Here I observed abandonment of agriculture/livestock. Here I observed abandonment of industry. Here I observed abandonment of housing. Here I observed expansion of areas for nature conservation. Here I observed expansion of agriculture/livestock. Here I observed expansion of industry. Here I observed expansion of abandoned areas. Here I observed expansion of housing. Here I observed restoration of natural areas. Here I observed restoration of public areas or specific buildings. If you won the lottery, what changes would you make in Colmenar Viejo, and where? If you had to defend at any cost a place subject to a change, which place would it be?

Respondents were allowed to map an unlimited number of places in response to each question. To ensure responses were located at a spatial scale adequate for analysis, a minimum zoom level for point placement was defined at level 14 (corresponding to a 1.35,000 nominal scale). No maximum zoom level was indicated (Table 2).

**Table 2:** Minimum and maximum zoom levels used by participants, associated map scales, and buffer distance used in the land use analysis

Zoom level	Nominal scale^a^	Buffer distance (m)
14	1:35,000	35
15	1:15,000	15
16	1:8000	8
17	1:4000	4
18	1:2000	2

^a^See http://wiki.openstreetmap.org/wiki/Zoom_levels

To provide supporting information about the study population, the following general statistical information was also solicited from respondents: (a) gender, (b) age, (c) number of years living in CV, (d) number of family members from CV, (e) whether respondents go out to the countryside with some family member and (f) whether respondents learn about the area with some family member. Finally, respondents were asked to express their level of agreement (strongly disagree, disagree, agree, strongly agree) with the following contextual statements;

- Nature is outside of the city.
- My family and I choose our consumer goods on the basis of price rather than origin.
- Environmental problems will be solved in the future thanks to technology and science.
- Landscape is cultural heritage but should be modified to satisfy the needs of the population.
- I take part in events, festivals and celebrations organized in Colmenar Viejo.
- I spend my leisure time after school at home.
- I am from Colmenar Viejo.

### Data Analysis

Spatial data were analyzed in GIS software and using the R software package (R Core Team 2016). The four research questions were used to construct further, detailed questions for data analysis, which was undertaken using contingency tables. Statistical associations between the variables in each table were examined using *X*^2^ and Fisher’s exact tests. For analysis of respondents’ mapped points and their relationship to land cover, we used CORINE land cover 2012 (CLC 2012) for overlay analyses (frequency, age and gender), and the Land Cover Information System for Spain (SIOSE) for land cover changes. To obtain an impression of the changes that had occurred in the study area we compared SIOSE data from 2005 with data from 2011 using cross tabulation (see, e.g., Pontius et al. 2004). In order to understand the role of distance in participants’ choice of locations, we also analyzed distances between the five participant schools and points chosen by respondents for questions relating to activities (place dependence) and emotions (place identity) in GIS and R software. For every point responding to each question the distance to the nearest school was calculated, and the predominant land cover type for each question was extracted. Results of the statistical analysis are discussed in the following section, and full results of all the statistical tests and data are provided in Appendix 2.

To aid the reader, contingency tables that showed significant relationships are visualized using mosaic plots, allowing raw data and Pearson’s standardized residuals (PSR) to be shown on a single plot instead of in two tables. The raw data are expressed using column width (columns data) and row height (row data). Widths and height depend on the number of points in each class. Residuals are coloured depending on whether there is a large positive difference between the observed value and the expected value (blue, evidence of association between rows and columns in that cell or attraction), a large negative difference between the observed value and the expected value (red, evidence of lack of association between rows and columns in that cell, or repulsion).

## Results

### Respondents’ Sociodemographic Background and Responses to Contextual Statements

A total of 747 respondents answered the questionnaire, corresponding to 52% male and 48% female. Respondents came from a wide variety of countries of origin, the majority from Spain (82%), with significant proportions from Ecuador (5%), Romania (3%) and Morocco (2%), and a wide range of other countries represented more than once, including most of the countries of the American continent (including the US), as well as China, Germany and the United Kingdom. Age of respondents varied between 12 and 18 years of age with an average (mean) age of 15. Almost three out of four respondents (72%) had lived in CV for at least 11 years; however, 41% of respondents said that they had no family member originally from the town. With regard to broader environment-related opinions, 65% of respondents agreed or completely agreed with the statement: Nature is outside of the city, 62% stated to limit their liberty to preserve voluntarily the environment (agree/completely agree). Fifty-eight percent of respondents and their families chose their consumption goods due to their price not their origin. Sixty-six percent of respondents believe that environmental problems will be solved in the future, thanks to technology and science. Respondents were not clearly positioned in relation to the changes in the landscape. 32% of respondents believed that landscape is cultural heritage but should be modified to satisfy the needs of the population, and 35% disagreed with this opinion.

### Results of Distance Analysis

The responses to the four directly natural environment-related questions (“In this place I collect nature products”, “Here I observed the environment’s ability to produce or renovate the air, soil and/or water”, “Here I perceive plants (flora), animals (fauna) or the ecosystem in general”, and “Here I enjoy the beauty of the landscape in general”) are all predominantly associated with grasslands, rather than urban fabric, and the average distances travelled to the selected locations from the nearest school are much greater for these explicitly natural environment-related responses than for the others. The shortest distances are for the two questions “Here I perceive local history” and “This place is emblematic for Colmenar Viejo”.

### Landscape Preferences and Change Perceptions by Question Type

#### Overall landscape preferences and change perceptions

**Fig. 2:**
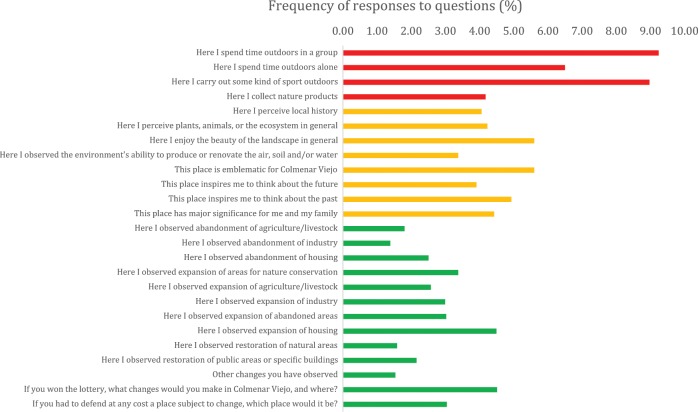
Frequency of responses per question: activities in red (top), emotions in yellow (middle), changes in green (bottom)

Total frequencies of points relating to activities, emotions and changes were quite similar for each of the three categories, with 1729 (29%) relating to activities, 2162 (36%) relating to emotions, and 2094 (35%) to changes. However, activities obtained higher frequencies of responses per question, since there were fewer questions (Fig. 2).The most frequently mapped points referred to “time outdoors in group” (9.2%), “carry out sport outdoors” (9%), and “time outdoors alone” (6.5%). After activities, emotions were the second most frequently mapped, with “beauty of landscape” (5.6%) and “emblematic for Colmenar Viejo” (5.6%) the most popular features in this category. In the changes category, “expansion of housing” (4.5%) and “if you won the lottery” (4.5%) were more frequently mapped than any of the other change aspects.

#### Association between questions answered and age

No correlation was found between the questions answered and the age of the respondents for any of the three categories.

#### Association between questions answered and gender

The four activities questions (spend time outdoors in group, spend time outdoors alone, carry out sport outdoors, collect nature products) elicited significantly different responses between males and females (*X*^2^ (3, *N* = 1716) = 8.18, *p* value = 0.04), with females preferring to spend time outdoors in group over the other three activities (PSR: *F* = 1.51, *M* = −1.43), and males preferring to practice sport outdoors (PSR: *F* = −1.30, *M* = 1.23). No correlation was found between gender and questions related to changes.

### Landscape Preferences and Perceived Changes by Land Cover

#### Overall landscape preferences and perceived changes by land cover

**Fig. 3:**
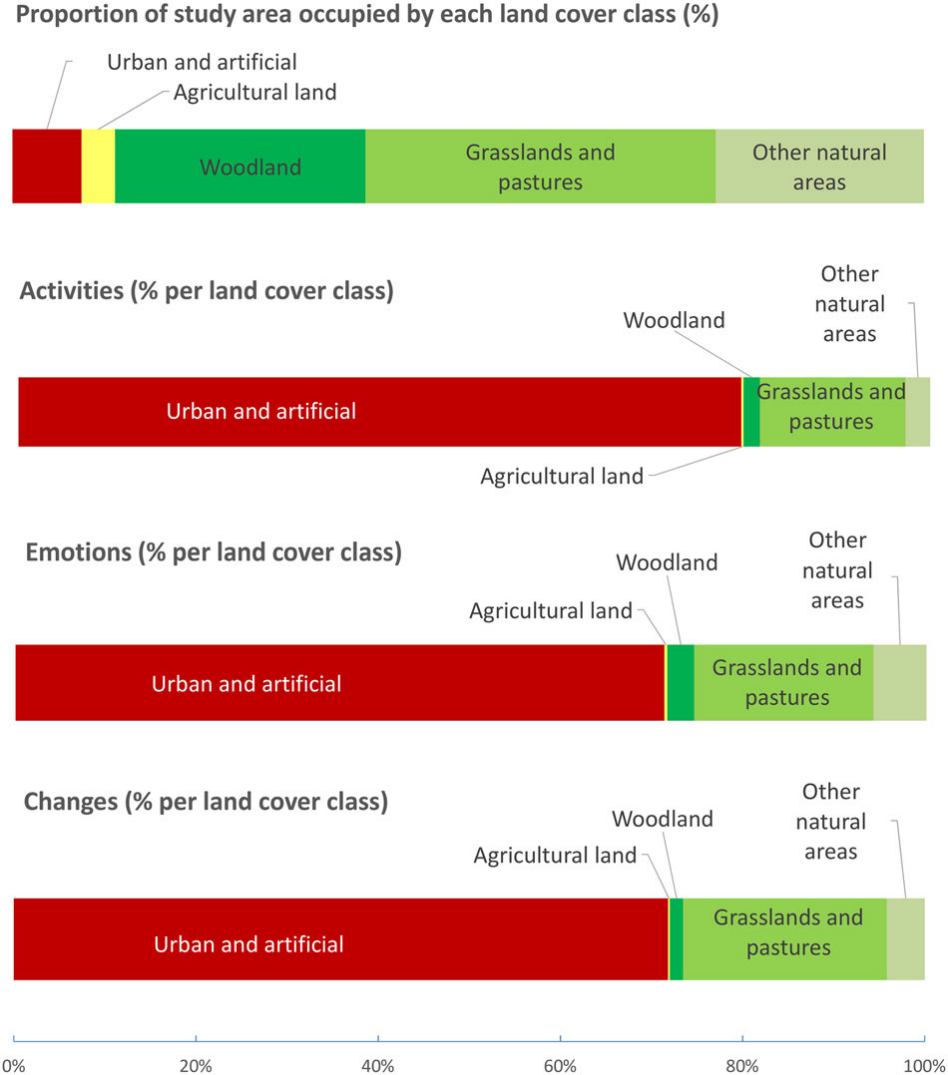
Percentages of activities, emotions and changes mapped by participants for each land cover type (class), and proportion of each land cover type in the study area

The majority of respondents’ points (74%) was located on urban and artificial land, in spite of the fact that this land cover type accounts for only 8% of the study area. The next most frequently occupied land cover type, grasslands and pastures (20% of respondents’ points), is poorly represented, despite the large number of respondents’ points mapped on this land cover. This is surprising because grasslands and pastures occupy 38% of the study area, and are easily accessible to the local population through an extensive network of cycling and walking trails. Other natural areas (4% of respondents’ points), woodland (2% of respondents’ points), and agricultural land (0% of respondents’ points) were even more poorly represented despite occupying 23%, 27% and 4% of the study area, respectively (Fig. 3).

#### Association between specific landscape preferences and land cover

**Fig. 4:**
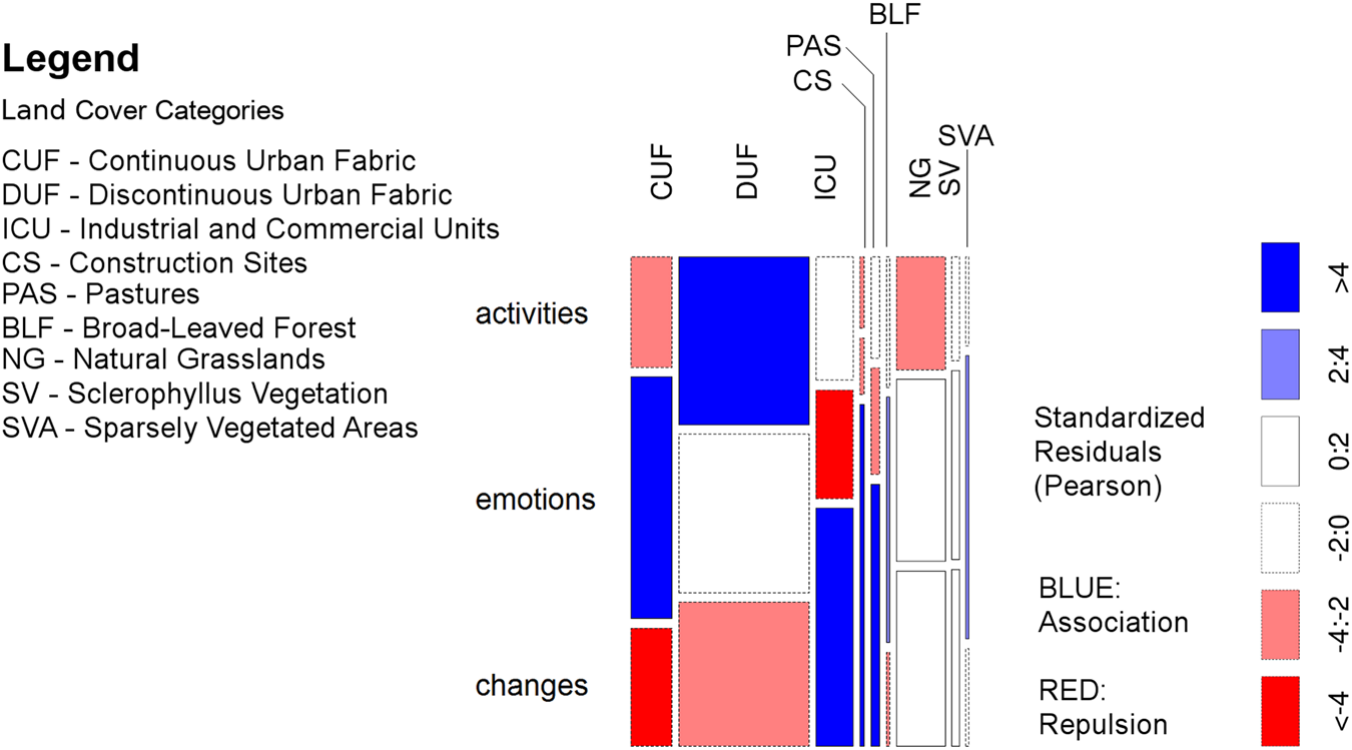
Mosaic plot of mapped points referring to activities, emotions and perceived changes by detailed land cover type. Blue indicates association between rows and columns in that cell, or attraction, Red indicates lack of association between rows and columns in that cell, or repulsion

The *X*^2^ test of association showed a significant relationship between land cover and the landscape category mapped by respondents: *X*^2^ (16, *N* = 5790) = 367.79, *p* < 0.001. Looking at the standardized residuals (Fig. 4), we can see clear patterns for activities, emotions and perceived changes to apply to particular land cover types.

Overall, activities were associated with discontinuous urban fabric—the dispersed residential suburbs—and negatively associated with natural vegetation or grassland (Fig. 4). Emotions were positively associated in particular with continuous urban fabric (the historic core of the city), but negatively associated with industrial and commercial units, construction sites and discontinuous urban fabric. Emotions were also positively associated with natural areas, in particular sparsely vegetated areas and broad-leaved forest. Changes, meanwhile, were associated with industrial and commercial units, construction sites and pastureland.

**Fig. 5:**
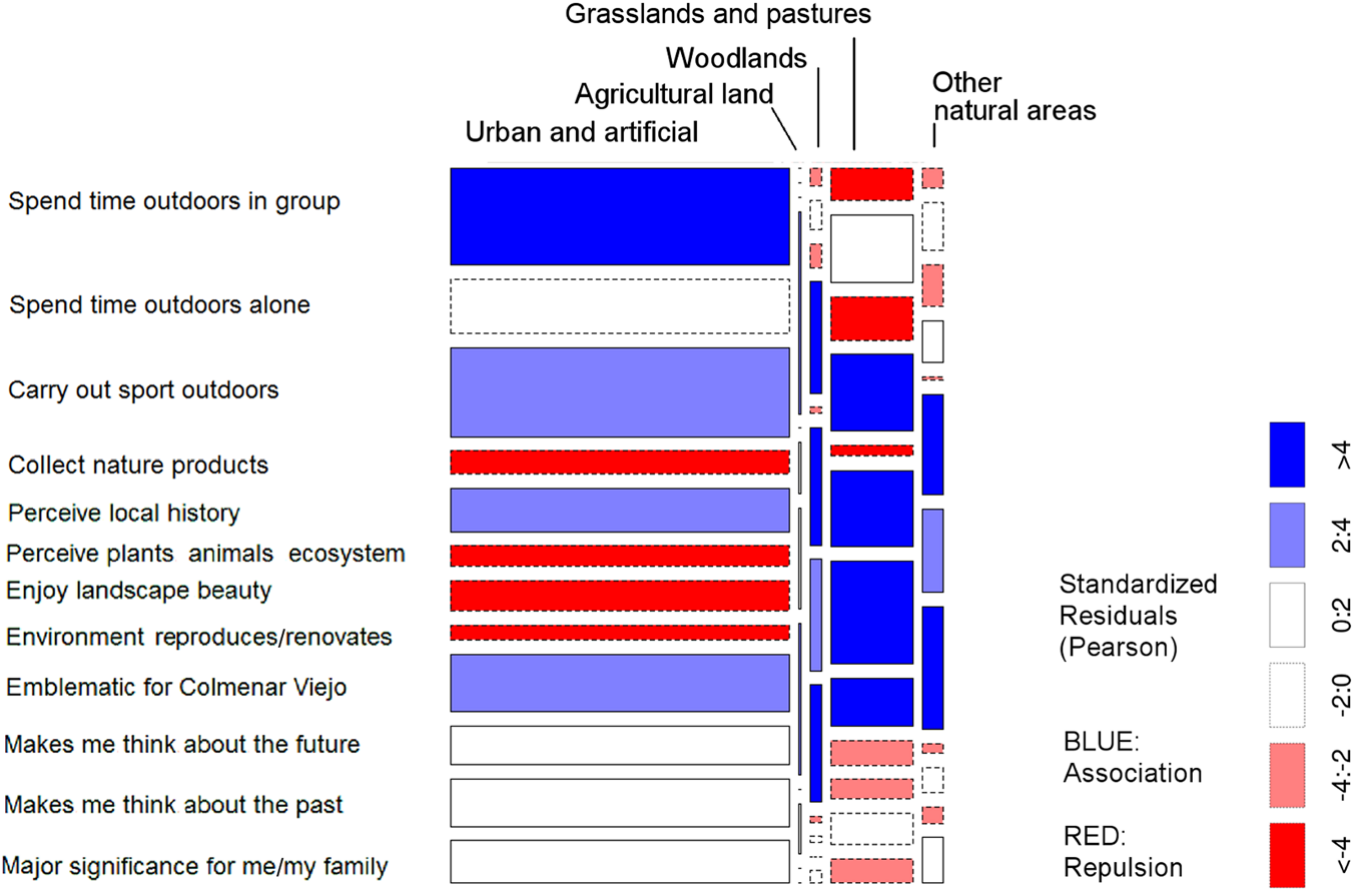
Mosaic plot of association between mapped points for specific activities/emotions and land cover type. Blue indicates association between rows and columns in that cell, or attraction, Red indicates lack of association between rows and columns in that cell, or repulsion

When activities responses were examined by specific question, the strongest positive association with urban land was observed for the “spend time outdoors in group” responses, with “spend time outdoors alone” negatively associated with urban land and “collect nature products” strongly negatively associated with urban land. “Collect nature products” was positively associated with woodland and grassland/pasture categories (Fig. 5). The mapped points for “carry out sport outdoors” were positively associated with urban areas and negatively associated with grasslands and pastures.

For emotions responses, the strongest positive association with urban land was observed for “Here I perceive local history”, and “Emblematic for Colmenar Viejo”. “Makes me think about the future”, “Makes me think about the past”, and “Major significance for me and my family” were likewise all associated with urban land cover types (Fig. 5). Responses for “Here I perceive plants, animals or the ecosystem”, “Here I enjoy the beauty of the landscape in general”, and “Here I observed the environment’s ability to produce or renovate the air, soil and/or water” were repulsed for urban land cover types, and strongly positively associated with woodlands, other natural areas and grasslands/pastures.

The association between responses for the questions related to perceived changes and land cover types are considered separately in Section “Adolescents’ Perception of Landscape Change Processes”.

#### Association between respondents’ age and mapped land cover type

A significant association between age of the respondents and land cover types was found for all three categories investigated, though the correlation was stronger for activities (*X*^2^ (15, *N* = 1580) = 47.21, p = 0.001) than for emotions (*X*^2^ (10, *N* = 1714) = 24.71, *p* = 0.006), or perceived changes (*X*^2^ (25, *N* = 1937) = 38.82, *p* = 0.038). Specifically:

- 13-year-old pupils were underrepresented for mapping changes for pastures.
- 14-year-old pupils were overrepresented in the group mapping activities on continuous urban fabric, but underrepresented for activities on natural grassland, and they also reported emotions relating to this land cover type less frequently.
- 18-year-old respondents were overrepresented for activities on natural grassland.

#### Association between respondents’ gender and mapped land cover type

A significant association between gender of the respondents and land cover type was found for activities (*X*^2^ (8, *N* = 1683) = 25.56, *p* = 0.001), but not for emotions (*X*^2^ (11, *N* = 2119) = 10.29, *p* = 0.504) or changes (*X*^2^ (8, *N* = 2031) = 12.13, *p* = 0.145), with significantly more males than females mapping activities on natural grasslands.

### Adolescents’ Perception of Landscape Change Processes

#### Landscape change in the period 2005–2011

The results of the cross-tabulation analysis served as a baseline to compare students’ perceptions of change with changes recorded by cartographic sources between 2005 and 2011 (Appendix 1). The SIOSE dataset is the most detailed available for this region, the 2011 map is the most recent available at the time of the survey, and performs well when compared to other datasets like CORINE Land Cover (see e.g., García-Álvarez et al. 2019; Tena 2011). It must be recognized however that no land cover dataset is perfectly accurate or suitable for every task in every case, and so should be treated as a broadly reliable guide, rather than a statement of absolute certainty. This aspect is discussed in more detail in Section “Limitations of the Study”.

**Fig. 6:**
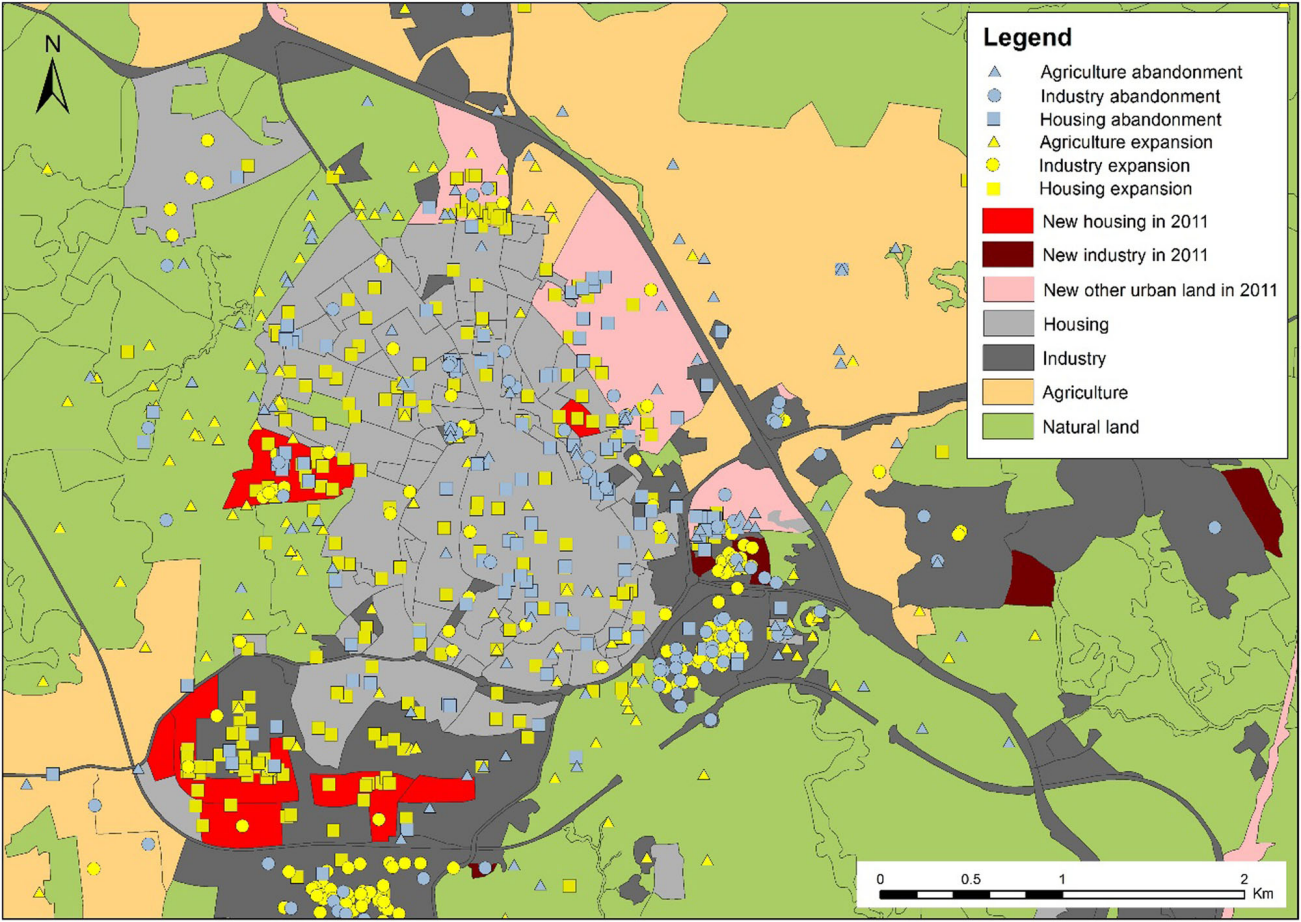
Land cover changes in Colmenar Viejo 2005–11 (SIOSE). Relationship of changes indicated by respondents (points) to mapped land cover changes

Out of a total of 63,324 ha in the study area, 929 ha (1.5%) changed from one land cover type to another during this period. The most important changes that took place relate to urban expansion (Fig. 6), with housing, infrastructure and new commercial and industrial developments occupying mostly former farmland but also brownfield sites, and woodland. Relevant to the analysis of landscape change perception, observed land cover change could be classified into two types, change related to expansion of some types of land cover, accounting for 865 ha (93%) of the total change area, and change related to abandonment of some types of land cover, accounting for 680 ha of change (73%) of the total change area. Total land change (969 ha) is not equal to the sum of the total area of expansion and abandonment (929 ha), because expansion of one land cover involves abandonment of another in the same location.

Five types of land cover expansion were identified, expansion of other urban land [not housing or industry] (320 ha, 37% of total expansion), expansion of industry (240 ha, 28%), expansion of housing (175 ha, 20%), expansion of agriculture (90 ha, 10%), and expansion of natural land (40 ha, 5%). Only two types of abandonment were detected from change analysis, abandonment of agriculture (460 ha, 68% of total land abandoned), and abandonment of industry (220 ha, 32%). No abandonment of housing or any other urban land cover type was identified.

#### Overall perception of changes

The *X*^2^ test of association showed a significant relationship between changes located by respondents and changes recorded by SIOSE (*X*^2^ (3, *N* = 1240) = 97.92, *p* < 0.001).

**Fig. 7:**
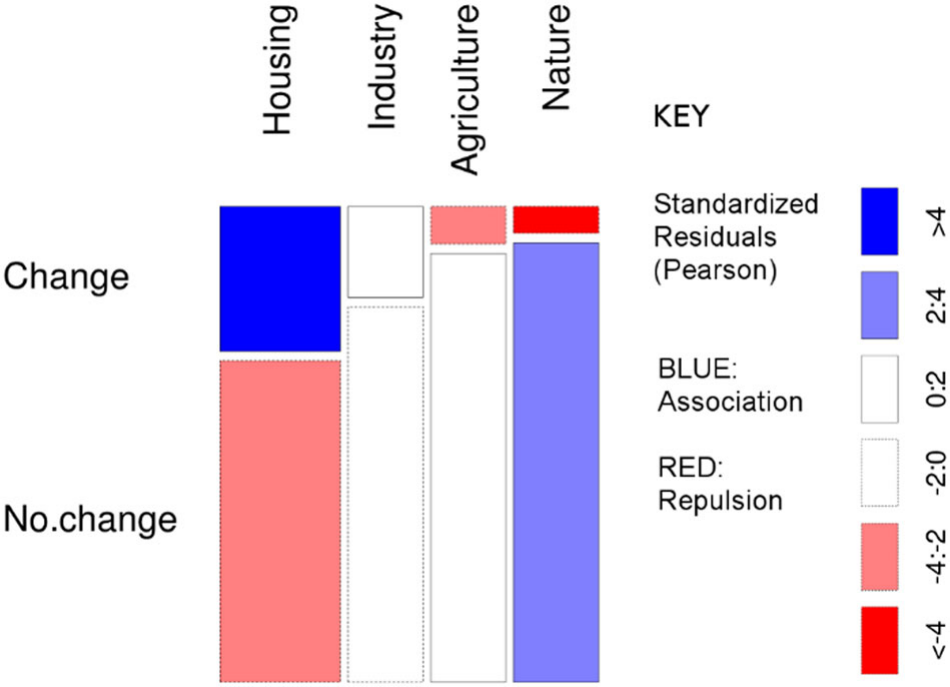
Mosaic plot of respondents’ mapped points by perceived change type vs. coincidence with areas of change 2005–11, and areas of no-change 2005–11

Examination of the standardized residuals (Fig. 7) however reveals a strong difference between urban areas and elsewhere in terms of association between changes mapped by respondents and changes recorded by the land cover maps. Locations of change to housing identified by respondents were strongly associated with areas where change to housing was recorded on the land cover maps. Locations of change to industry identified by respondents were weakly associated with areas where change to industry was recorded on the land cover maps. There was a strong negative association between locations of change to agricultural and natural areas identified by respondents and change to these categories recorded on the land cover maps.

#### Perception of changes relating to expansion

**Fig. 8:**
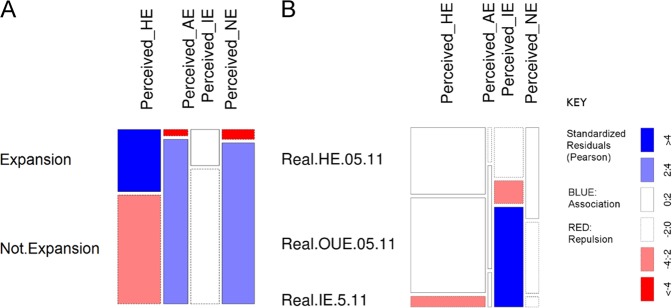
**a** Mosaic plot of respondents mapped points by perceived change type vs: **a**: coincidence with areas of expansion 2005–11, and areas of no expansion 2005–11; **b** coincidence with different types of land expansion. HE housing expansion, AE agricultural expansion, IE industrial expansion, NE nature expansion

Respondents identified 804 locations as relating to expansion of agriculture/livestock, industry, housing or nature. Of these 154 (19%) coincided with areas where expansion was detected from analysis of land cover maps. Though this seems like a low percentage, it is significantly better than would be expected by chance (X^2^ (3, *N* = 804) = 98.28, *p* < 0.001, Fisher’s exact test (*N* = 804, two sided), *p* < 0.001). This was mostly due to the high level of coincidence between respondents’ perceived expansion of housing, and locations that had experienced some kind of change (though often not housing) (Fig. 8a).

Association between respondents’ points and mapped changes for different types of land expansion was less strong than for the simple expansion vs no expansion case above, but still stronger than would be expected by chance (*X*^2^ (6, *N* = 158) = 54.02, *p* < 0.001, Fisher’s exact test (*N* = 158, two sided), *p* < 0.001). There was much stronger association between respondents’ points for industry expansion and industry expansion detected by analysis of land cover maps than for all other kinds of expansion (Fig. 8b).

#### Perception of changes relating to abandonment

Respondents identified 341 locations where they believed that abandonment of agriculture/livestock, industry or housing had taken place (Appendix 1). Of these, 57 (17%) coincided with areas where abandonment was detected by analysis of land cover maps. However, in this case, a significant association could not be detected between respondents’ perception of abandonment and real abandonment (*X*^2^ (2, *N* = 341) = 2.09, *p* = 0.35, Fisher’s exact test (*N* = 341, two sided), *p* = 0.38). In other words, chance alone could explain the fact that 57 points related to abandonment fell on areas that had really been abandoned.

#### Evaluation of future changes

There was no correlation between the age or gender of respondents and the frequency of answers to the future change questions: “If you won the lottery, what changes would you make in Colmenar Viejo and where?” and “If you had to defend at any cost a place subject to a change, which place would it be?” Places that would be defended at all costs included two parks, a school, the sports complex (Polideportivo Martín Colmenarejo), and the bullfighting arena (Plaza de Toros), as well as the natural area of Las Cuevas (threatened by encroaching urban development), and the allotment gardens at La Bastiana. Places that respondents would change if they won the lottery were concentrated around the sports complex and bullfighting arena, for reasons including revival of bullfighting, restoration of the Plaza or reuse for other purposes. However, the largest concentration of points relating to this question was situated on the Ventanal de la Sierra shopping mall, reflecting respondents’ desire for additional clothing/fashion stores and a cinema. When responses from the two questions relating to future changes at each of these locations were classified by age and gender, samples were too small to allow correlation analysis of these variables, with one exception: males and females for the Ventanal mall and the bullfighting arena. However, no significant association between gender and choice of either of these locations was found (*X*^2^ (1, N = 46) = 0.4, *p* = 0.52).

Unsurprisingly, given the observations made above about respondents’ preference for certain locations, a significant association was detected between land cover type and the questions on future changes (*X*^2^ (2, *N* = 446) = 8.39, *p*-value = 0.02). Answers referring to the “If you won the lottery” question were slightly associated with urban and artificial land (PSR 0.65) and repulsed for grasslands and pastures (PSR −0.65) and other natural areas (PSR −1.58). Conversely, responses on the “If you had to defend at any cost” question systematically and strongly coincided with other natural areas (PSR 1.94), slightly coincided with grasslands and pastures (PSR 0.8) and were repulsed by urban and artificial land (PSR −0.8).

### Precision in Spatially Identifying Landscape Preferences and Change Processes

**Fig. 9:**
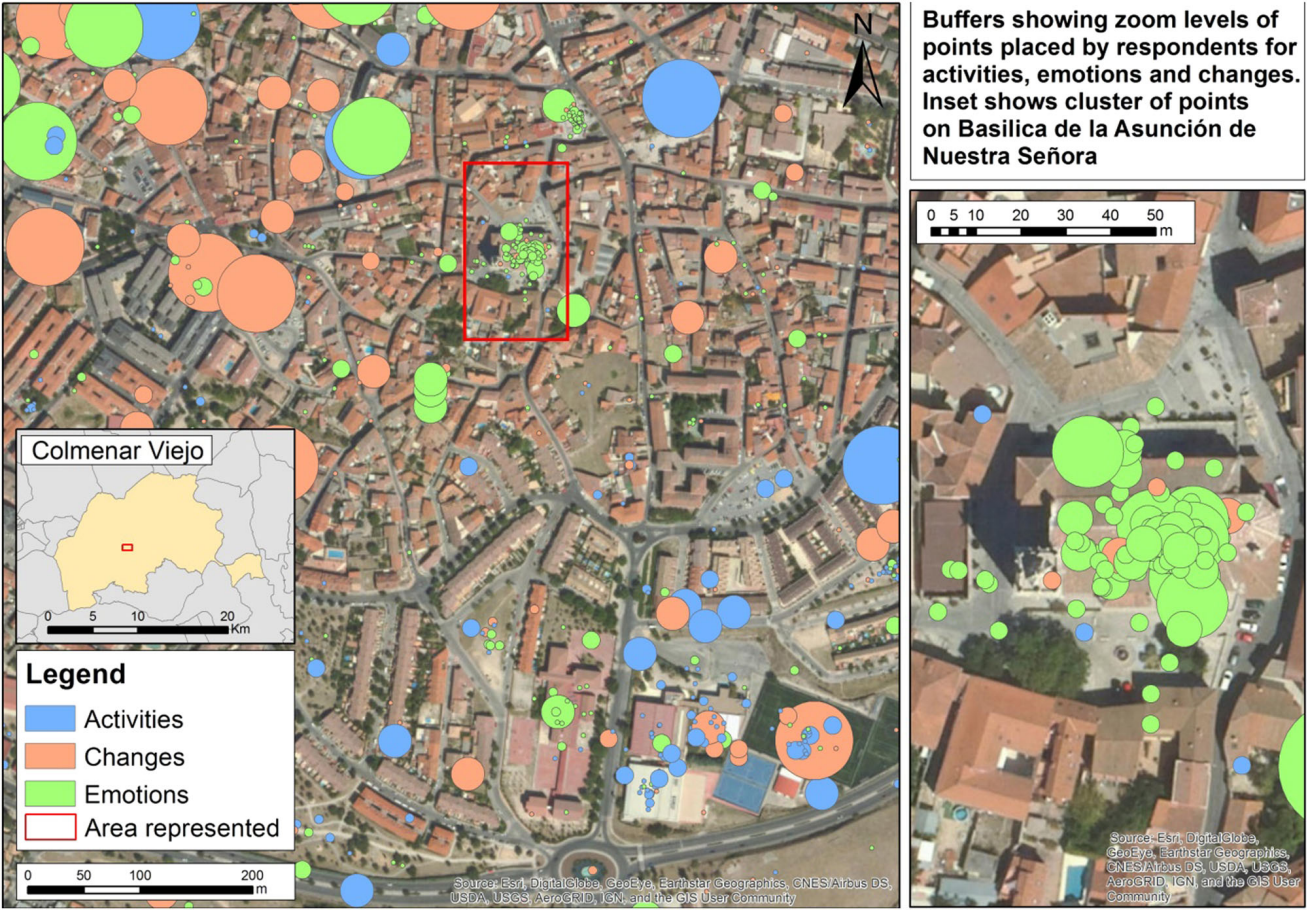
Example of points placed by respondents representing landscape preferences and perceived change processes with circles of “buffers” illustrating the zoom level at which they were placed. The radius of the buffers corresponds to scale of each zoom level, and thus reflects the level of uncertainty in point placement

The ability to zoom in and out of digital map layers offers respondents the ability to locate elements with varying degrees of precision (and by extension; accuracy), and we were interested to know if this capability expressed itself systematically such that particular landscape change perceptions and preferences were routinely located more or less precisely than others (Fig. 9). The *X*^2^ test of association showed a significant relationship between the zoom level chosen by participants and landscape preferences and perceived changes (*X*^2^ (8, *N* = 6316) = 257.36, *p* < 0.001).

**Fig. 10:**
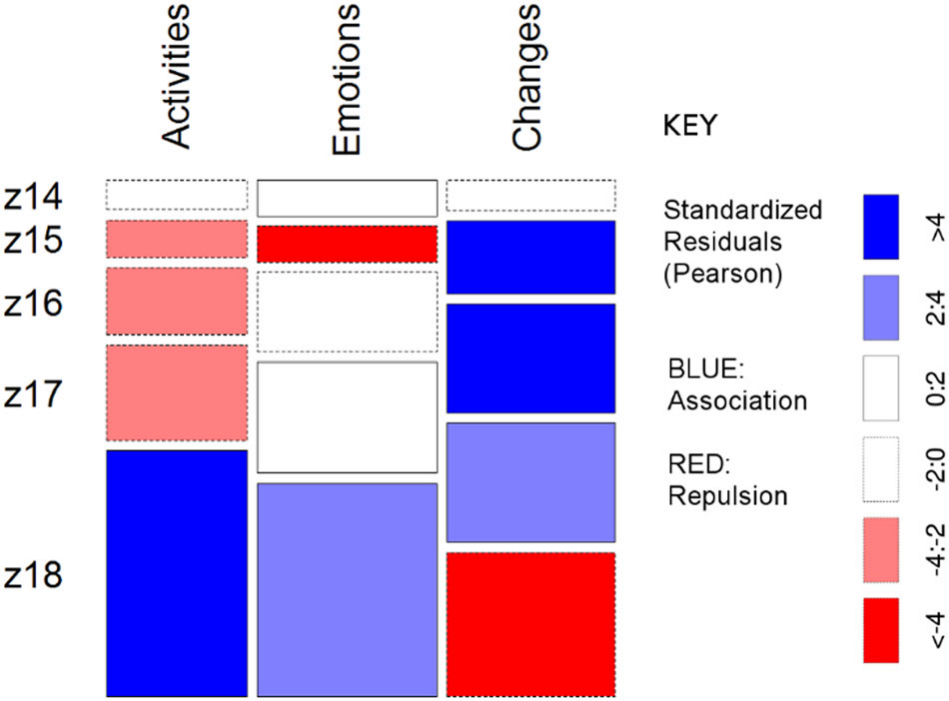
Mosaic plot of standardized residuals for zoom levels at which respondents located activities, emotions and changes

More than 50% of the points that indicate locations where respondents carry out activities were placed with zoom level 18, the largest, i.e., most detailed, zoom level available, so the intention of pinpointing specific locations can be intuited. The rest is distributed between zoom levels 14, 15, 16, and 17, with ~6%, 8%, 14% and 20% of the points, respectively. Test results show that activities were mapped significantly more precisely than expected, with zoom levels smaller than 18, indicating less-precise pinpointing, significantly underrepresented (Fig. 10).

With respect to emotions, ~45% of the points that indicate locations that arouse emotions in respondents were also placed with zoom level 18, that is, in great detail. Zoom levels 14, 15, 16 and 17 received about 7%, 7%, 17% and 23% of the points, respectively. Although locations that are connected to respondents’ emotions are also very precisely located, the responses show more general variance than in the case of activities. Zoom level 18 was significantly overrepresented, and Zoom level 15 significantly underrepresented (Fig. 10).

Respondents’ points relating to changes were evenly distributed across the three largest zoom levels: ~30% of the points were placed with zoom level 18, 25% with zoom level 17, and 23% with zoom level 16. However, a look at the standardized residuals (Fig. 10) shows that the changes are mapped significantly less precisely than expected, at zoom levels 15 and 16 rather than at zoom level 18.

## Discussion

Correlation between respondents’ activities and different land cover categories brings to light different aspects of sense of place in the adolescents’ everyday place experiences in the context of a rapidly changing peri-urban landscape. In the following section, we discuss what we feel to be the most important findings in the light of sense of place literature.

### What Do Landscape Preferences Tell Us about the Adolescents’ Sense of Place?

The points mapped by respondents were overwhelmingly located on urban land, with natural and agricultural land cover types underrepresented. However, closer examination allows a more nuanced picture to emerge.

For activities, it is those that were undertaken outdoors in a group which were the most strongly associated with urban land, followed by carrying out sport outdoors, with grasslands and pastures being preferred for spending time outdoors alone and collecting nature products. However, the overall picture is imbalanced because the two questions which elicited mostly urban areas-related responses gained 63% of the total number of answers for all four activities questions. “Here I spend time outdoors in a group” received five times as many responses as “Here I collect nature products”. This striking imbalance may be because social activities and organized sport events in the urban periphery, close to respondents’ homes and schools, is a common feature of the lives of most adolescents.

**Fig. 11:**
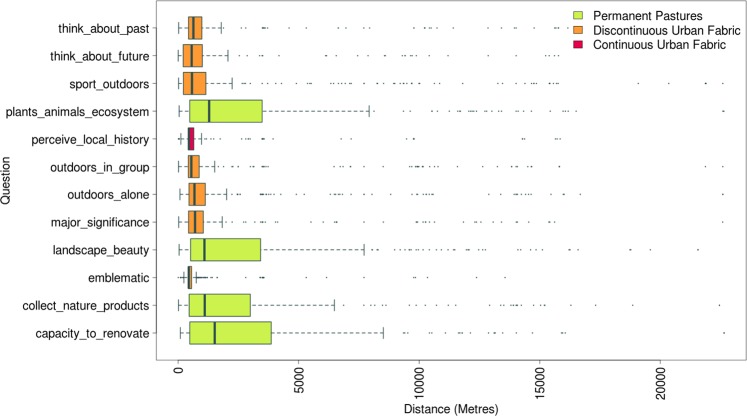
Distances to nearest school for all responses to activities and emotions questions, coloured by predominant land cover

The distance analysis (Fig. 11) sheds further light on this aspect. The separation between natural environment-related questions (all predominantly associated with grasslands) and the very small distance range for the responses “Here I perceive local history” and “This place is emblematic for Colmenar Viejo” indicate a highly localized sense of place identity. Locations preferred for “Spending time outdoors in group” are also mostly at short distances from participants’ schools—typically no further than ca. 1 km—which would seem to indicate a small distance range for social activity (see below). At the same time, the outliers indicate that at least some activities were undertaken at distances accessible only by car—all responses include several preferred locations up to 17 km away from the town, and occasionally at distances greater than 20 km. Though it is not possible to be certain, the impression is that these adolescents enjoy some localized freedom within the town, punctuated by occasional (probably motorized) trips further afield. This may reflect a growing preoccupation in modern developed countries for the safety of children, and the desire to have them close at hand, or undertaking structured activities, potentially depriving them of what Jack (2010) has referred to as “the freedom to use local areas in a relatively unstructured way and to visit favourite places and people independently”. However, it also true that the town of CV is not large, and predominantly surrounded by pastures, so the impression given by Fig. 11 of a local range of around 1 km may simply relate to the physical characteristics of the town and the limited access of this age group to motorized transport. These results offer an interesting point of comparison with other studies of adult residents’ “sense-in-place” in terms of distance, e.g., Cantrill and Senecah (2001), who found marked differences between the area that respondents’ called home in two separate US towns—less than 2 miles (3.2 km) in Palatine, IL; against ~10 miles (16 km) in Marquette, MI. Probably, factors such as urban design (compact core city vs. sprawling suburban development) and access to motorized transport (less access for children, the elderly and socioeconomically marginalized groups) would need to be accounted for in future studies of distance-related sense of place.

While the two activities questions that were associated with grasslands and pastures (“Here I spend time outdoors alone” and “Here I collect nature products”) received fewer responses than those for urban land, they are also quite revealing. While the association of nature products with grasslands and pastures and woodlands seems logical, the preference for these land cover types for spending time alone is significant, possibly implying feelings of city exclusion (Malone 2002; cited by Abbot-Chapman and Robertson 2009), and suggesting a desire or need to become immersed in local semi-natural or natural areas as a means to escape the pressures of daily life. Stewart (2003) notes the importance of including immersive outdoor experiences, giving time to experience nature, rather than just rushing in and out to carry out activities, in children’s education.

For the emotions category, the answers to the three questions specifically relating to the natural environment (“Here I observed the environment’s ability to produce or renovate the air, soil and/or water”, “Here I perceive plants (flora), animals (fauna) or the ecosystem in general” and “Here I enjoy the beauty of the landscape in general”) showed strong tendencies to be located on grasslands, woodlands and the other natural areas, with the exception of the question about landscape beauty, which was more strongly associated with grasslands and pastures than the other two nature categories. This apparent preference for grasslands and pastures over woodlands or other natural areas seems surprising, and leads one to wonder whether grasslands are more accessible than other natural areas, or whether the pastoral roots of the local economy are presented in the collective imagination.

Further, different types of urban land also elicited different responses. Responses in the emotions category—related to history (“Here I perceive local history”, “Here I am inspired to think about the past”), local identity (“emblematic for Colmenar Viejo”) and personal identify (“significant for me and my family”)—were associated with the core city, and, in particular, with important historic buildings like the 16th century Basílica de la Asunción de Nuestra Señora or the Town Hall. These buildings are a strong part of the town’s cultural and historical identity, and a key part of its marketing, appearing in most of the images and representations of the town in the media—magazines, websites, products, etc.

On the other hand, the dispersed urban suburbs (land cover type “discontinuous urban fabric”) are seen as functional yet unemotional locations, where respondents carry out most of their activities, while industrial areas are less associated with emotions than any other land cover type, being viewed mostly as locations of change. Indeed, large amounts of industrial land have been recently transformed into housing. The association of perceived changes with industrial and commercial units, construction sites and pastureland, may reflect the strong impressions left on the adolescents by new urban development, which has predominantly taken place on these land cover types. This may be due to these being the most visible short term-changes in this area.

While the two activities questions “Here I carry out some kind of sport outdoors” and “Here I spend time outdoors in a group” were both associated with the dispersed urban suburbs away from the historic core, there is some evidence of the distinction noted by Kyle et al. (2005), between locations that are preferred because of their functional value, i.e., a place where a particular activity can be carried out, and locations that are chosen for their social meaning. The association between the sport question and the urban periphery is likely to be related to the location of sports facilities—although responses did include some sports that could be carried out in the countryside, like running, walking and cycling, the majority of the sports referred to required dedicated facilities (athletics, rugby, tennis, volleyball, handball, basketball, football etc.). This pattern is an example of functional place dependence, and shows the importance to local populations of these suburban sports facilities which are closely associated with the urban expansion phase of the last two decades. On the other hand, the responses for “Here I spend time outdoors in a group” show a pattern that may be more closely related to social bonding—what Kyle et al. (2005) refer to as “locations and settings as a context for relationships and shared experiences”—than the location of specific installations. For example, a small cluster of responses to this question (23 of 553 responses) was identified within the Ventanal de la Sierra shopping mall. The idea that the shopping mall may be more important for social bonding than for specific activities is reinforced by the fact that this was also the most frequently identified place that respondents would change if they won the lottery (Section “Evaluation of future changes”). Adolescents’ preference for meeting in shopping centres or malls on the urban periphery is well documented in Anglo-American societies (see e.g. Matthews et al. 2000). These malls are a comparatively recent urban phenomenon in Spain (Alemán and Díaz 2006), and have proliferated in recent years as part of the urban development boom of the early 2000s (Díaz-Pacheco and Hewitt 2010). However, this distinction between functional locations and social bonding locations should not be overstated due to the potential overlap between the two - the mall is clearly a functional location as well as a meeting place, and sports activities are a typical opportunity for social bonding.

### Age, Gender and Landscape Preferences

Adolescents’ perception of landscape evolves in alignment with the experience lived through activities and emotions. This is how sense of place is shaped. Thus we would expect to find clear differences between older and younger people. Interestingly, the preference shown by 14-year-olds for the town centre, is also found in a study of adolescents’ favourite places in Tasmania, Australia (Abbot-Chapman and Robertson 2009). This study suggests that this may be because younger adolescents may not yet have begun to experience feelings of city exclusion described by Malone (2002; cited by Abbot-Chapman and Robertson 2009). It is unclear, however, if the centre of a small town like CV is really comparable to the Australian urban environment documented by this author.

The preference of 18-year-old respondents for carrying out activities on grasslands, compared with the tendency of younger adolescents to actively avoid this land cover type in preference for town-based activities may also be a reflection of the process of maturity, where the adolescents’ focus shifts away from the home towards places and activities in the local area as they grow older, a pattern also observed in the Tasmania study (Abbot-Chapman and Robertson 2009). Older adolescents are likely to roam more widely outside of the family home than younger ones and the eldest respondents may have a motor car driver’s license (minimum age 18, though mopeds can be driven in Spain from the age of 15, and light motorcycles from 16).

With regards to gender, males were significantly more likely to carry out activities on grasslands than females. There are several possible explanations for this. Males may be more disposed to carry out outdoor activities generally than females, noting that 68% of respondents said that they went out into the countryside with a male, rather than female, family member. This tallies with Mäkinen and Tyrväinen’s (2008) study of teenagers’ uses and values for urban green space in Helsinki, where girls used public green spaces less than boys and preferred walking while boys preferred more strenuous activities like cycling or playing football. Abbott and Robertson (2009) found that girls tended to choose home and own bedroom as their “favourite places” more than boys, who tended to choose places away from the home. Females may be considered more vulnerable than males, and males may be allowed out on their own from a younger age. The preference shown by females for the “spend time outdoors in group” response over the other four activity options, a pattern not seen for male respondents, lends support to these inferences.

### Adolescents’ Perception of Landscape Change

Changes to housing identified by respondents were strongly correlated with changes recorded by the cartographic sources. The most frequently perceived change mapped by respondents was “housing expansion”, and these changes were mapped accurately when compared with land cover maps. However, no correlation was detected between changes to agricultural or natural land identified by respondents and this type of change as recorded by the land cover maps. This may suggest that respondents are not well-attuned to non-urban aspects of their surroundings. However, it should also be considered that growth of housing and other urban areas has been very significant, as the land cover analysis confirms (Appendix 1) and urban development is more striking than gradual loss of natural or semi-natural areas.

With regards to expansion processes of certain land cover types, there was good evidence that adolescents are highly sensitive to urban land expansion in their immediate surroundings. The survey is not able to show whether they felt these elements to be positive or negative in general, but the evidence of a strong link between their perceptions of change and real observed change suggests that these changes are important enough to them to have been memorized with a high degree of accuracy. The responses to “defend at any cost” question indicate awareness of the negative aspects of urban expansion on at least one favoured location (Las Cuevas).

However, respondents’ mapping of abandonment processes did not correlate with areas of abandonment recorded by the land cover maps. This may be due to inaccuracies in the land cover map, or it may be that participants did not share a single clear understanding of what this meant, and were not able to clearly associate the concept with the disappearance of particular land cover types through transformation into others. The interpretation of loss to particular land cover categories as abandonment is in any case debateable, so this result is not surprising.

### Implications for PPGIS Methodology

PPGIS studies frequently employ analysis of spatial clustering (Brown and Fagerholm 2015). This did not seem appropriate in our case. This is because clustering is dependent on scale, and our points had been placed at multiple scales, as a result of the ability to zoom in and out of digital map layers, and therefore to locate elements with varying degrees of precision. While potentially helpful for respondents, it significantly complicates the analysis, since the resulting data layer of responses, unlike traditional cartographic datasets, was effectively a superimposition of objects of differing scales. However, while spatial cluster analysis could not be reliably undertaken for this reason, the dataset did offer an opportunity to analyze the relationship between zoom level and the elements mapped by respondents in each category of responses. In fact, this tendency seemed to have expressed itself systematically with activities and emotions located more precisely than would be expected by chance. Changes, on the other hand, were mapped less precisely than would be expected by chance. Perhaps respondents wished to locate a general area where change had occurred, not a specific point. Another possible explanation would be that respondents were quite familiar with change locations and did not need to zoom in so far to identify them. Alternatively, the opposite might be true—use of a less-precise zoom level might indicate uncertainty (“I think it’s somewhere around here”). More detailed exploration into respondents’ mapping habits would be needed to clarify these tentative suggestions for respondents’ choice of zoom level.

In general, the multi-scale nature of such datasets is problematic for analysis of spatial clustering. There seemed to be no secure way of identifying a meaningful spatial cluster in such a dataset through any standard statistical approach. As these kinds of problems are likely to be common in PPGIS surveys, we draw attention to this issue as something worthy of further attention in future research.

### Points of Comparison with Other Recent PPGIS Studies in the Madrid Region

It is instructive to compare the results of the present study with two recent participatory mapping studies from the Madrid region. In the first of these, García-Martín et al. (2017) apply a PPGIS approach similar to that employed here to investigate landscape values in a pan-European study that included a sample population from CV. In a second study, Pérez-Ramírez et al. (2019) use a participatory mapping approach to investigate sense of place on agricultural lands in a cultivated region to the southeast of Madrid (our own study area lies to the north, on largely pastoral lands). The results of both these studies offer interesting points of comparison.

The first study (García-Martín et al. 2017) sampled an adult, rather than adolescent, population in CV, did not focus on landscape changes, and employed a different statistical approach to data analysis that used here. Both similarities and differences emerge between this study and our own.

In terms of similarities, the very high point densities on urban land recorded by García-Martín et al. (2017) is clearly analogous with the findings of this study, in which urban land was overwhelmingly preferred by respondents for all three landscape aspects. Further, just as in the García-Martín et al. (2017) study, the most frequently mapped landscape preferences in our present survey were related to outdoor sports and recreation. However, in the earlier study, most places connected to outdoor recreation activities were mapped on natural grasslands. In the adolescent group sample, outdoor sports were negatively associated with this landscape type, and overwhelmingly mapped in the urban area, coinciding with dedicated sports facilities.

These authors also found that the oldest group of respondents identified more landscape activities and emotions than younger respondents (García-Martín et al. 2017). The authors posed two possible interpretations for this: (1) that perception of landscape values increases as people grow older, or (2) as a result of generational change, with younger generations in general being less connected to landscapes than older generations. The results of the present study lend to support to the first interpretation, since older students identified more landscape preferences connected to outdoor recreation activities outside the urban area than younger ones. However, this could also be due to the fact that only the older students are independent enough to venture outside of the urban area for their activities.

The most important difference between García-Martín et al. (2017) and the current study is that while the earlier work was framed in terms of ecosystem services (Millennium Ecosystem Assessment 2003), the focus of the present work has been on land cover changes. As a result, the insights obtained here shed more detailed light on the specific landscape types favoured by respondents, as well as the accuracy of their perception of change in the context of rapid transformation in a fast-growing region (see Hewitt and Escobar 2011).

In the second study considered here, Pérez-Ramírez et al. (2019) investigated the sense of place on cultivated lands in the Las Vegas agrarian district to the southeast of Madrid, through a participatory process that included participatory mapping activities. The participant sample was drawn from the local population including farming professionals, land-use decision makers and planners and other local actors with interest and involvement in agricultural lands. Participants shared their knowledge on former agrarian practices and traditions, mapped the location of past and present cultivated lands and places of special value to them, and envisioned future land use changes through a scenario planning approach.

The most notable difference between this study and the present one is the Las Vegas respondents’ clear preference for cultivated lands. Of the locations valued by participants as important to them in some way, 65% overlapped with cultivated land areas, as opposed to only 15% on non-natural, non-productive land mostly urban or other artificial land cover types. The contrast between the dominant urban land preferences of our own study is striking. It would be interesting to see if adolescent populations in the Las Vegas area also valued cultivated areas, or if they followed the more urban preferences of the adolescents in our own study.

In terms of respondents’ preferences for future land-use pathways, natural areas, green and protected areas and horticulture areas were the most highly desired (Pérez-Ramírez et al. 2019). This stands in contrast to our own study, where adolescent responses to the “if you won the lottery” question were associated with urban and artificial land.

### Implications for Cultural Heritage and Urban Planning

Analysis of the distribution of responses among the different urban classes is able to reveal a striking distinction between the urban core area and the suburbs. In CV, as in much of Spain, this urban-suburban boundary is very clearly defined, a consequence of the very recent expansion of sprawling peripheral developments which have more in common with Australian or US cities than the towns of historic Castile. The adolescents’ responses are a direct reflection of this physical separation of urban space and are a clear testament to the increasing focus of activities like sport and shopping in the urban periphery, with the urban core retaining its importance for emotional or aesthetic aspects of the human experience, like historical memory and sense of place and belonging. This finding supports recent calls for broader narratives around cultural heritage that are able to account for these intangible experiences beyond just recreation and tourism (García-Martín et al. 2018). Moving away from the idea that “that which has no market value has no value at all” (Díaz-Pacheco and Hewitt 2010) towards new kinds of spatial planning that can accommodate these intangible landscape values is in any case likely to reap wider economic benefits in the long run. A visitor to CV might remark on the contrast between the rather lacklustre commercial activity in the urban core and its bustling periphery, and wonder whether an alternative town planning approach might seek better integration of the town’s economic activity with the cultural heritage spaces of the urban core.

### Limitations of the Study

A number of limitations can be identified in our study. First of all, there is potentially some urban bias in the questionnaire itself, the clear focus on landscape notwithstanding, due to the orientation of the study around CV, which is an important service centre, despite its rural location. In support of this hypothesis, in the study by Pérez-Ramírez et al. (2019), urban areas were in the minority of places valued by participants (unproductive lands, 15%, Fig. 3 in Pérez-Ramírez et al. 2019). This is probably because, in that study, the sense of place mapping activity took place in context of a workshop on cultivated lands, and directly after an activity about areas of agricultural importance and current cultivation. Thus, an element of bias towards agricultural areas in that study could be reasonably claimed, and an element of bias towards urban areas in our own study cannot be entirely excluded.

Against this hypothesis, however, it can be noted that many of the questions in our study did encourage respondents to think specifically about natural areas, and we should be cautious in rushing to categorize this group of adolescents as narrowly focussed on exclusively urban matters. Yet it is true that CV is a medium-sized modern town, not a village, in a region whose economy is dominated by services, not agriculture, and respondents’ perceptions are likely to reflect this.

Further, the conclusions one is able to draw are necessarily an artefact of the questions asked and there is some ambiguity. For example, a focus on sport seems to emerge, but it is not clear whether all participants considered enjoying the countryside by walking or cycling to be a sport. A separate question related to enjoying the countryside (as distinct from appreciating landscape beauty) might have helped clear up this doubt.

A further limitation relates to the unavoidably wide range of uncertainty in the datasets, due to the differences between mapped scale of responses and scale of the land cover dataset, respondents’ uncertainty in location, the difference between the most recent available map and the date of the survey (ca. 5 years) and inaccuracies in the land cover dataset. With respect to this last question, these inaccuracies were minimized by using the SIOSE, rather than CORINE land cover, for analysis of changes at the parcel scale, in line with recommendations of a number of recent authors (Díaz-Pacheco and Gutiérrez 2014; García-Álvarez and Olmedo 2017). However, even in the SIOSE dataset, limitations have been recorded in the identification of some land cover types (see e.g., Díaz-Pacheco et al. 2018). Mixed land uses in urban areas are notoriously difficult to accurately map, and this may explain some discrepancies between changes identified by respondents and the land cover map. For example, the difficulty experienced by respondents in separating housing from other urban land may be because the “other urban” land cover category, which refers to urban land not specifically designated as housing or industry, e.g. shops and offices, may contain some housing, known to respondents, but not recorded as such in the SIOSE dataset. It is also true that respondents were not asked to locate expansion or abandonment of shops and offices, just housing, industry, agriculture and nature, so they may have included expansion of commercial or mixed-use premises as “housing”. The strong association between changes perceived by respondents and changes detected from SIOSE between 2005 and 2011 is reassuring in view of these uncertainties.

## Conclusions

The adolescents engaged in this study are probably broadly representative of adolescents from middle income backgrounds across Spain, particularly those in medium-sized towns in rapidly urbanizing regions. They are highly social, preferring to do things together in groups rather than alone, and strongly focussed on activities, especially sport, which they habitually do in designated sports complexes and usually away from the urban core. This contrasts with the adult population from the same town, who strongly associated outdoor sport and recreation with grasslands, rather than urban areas (García-Martín et al. 2017). This is probably a consequence both of the fact that awareness of landscape develops with age (as supported by various studies) as well as the rapid changes that have occurred in the town in terms of development of new installations and facilities that adults had not previously enjoyed.

Despite the high foreign-born percentage in the sample population, they have a strong sense of local identity, suggesting a fairly adaptable and well-integrated group. They are environmentally aware, and many respondents felt a strong emotional attachment to the town’s public spaces and historic sites. In this part of Castile, traditional Spanish cultural activities like bullfighting are valued by adolescents alongside visiting nearby malls for shopping and leisure. Their interest in the municipal allotment gardens at La Bastiana is also another indicator of continued importance in Spanish society of small-scale horticulture for leisure and subsistence.

There was a notable tendency for respondents to carry out activities outside of the historic core, but to retain emotional or cultural attachment to places within it, no doubt reflecting the urban layout and the implicit and explicit separation of “functional spaces” from “historic spaces”. This sharp dichotomy may suggest a lack of integration of cultural heritage in spatial planning. It is also a reflection of the very well-known tendency for services and activities to relocate to the urban periphery, depriving the urban core of economic diversity, and ultimately, its urban identity, a transformation process that is well documented in Spain (e.g. Méndez 1994). That this distinction emerges so clearly in the use of space by the town’s adolescents suggests that better integration of the historic core and the periphery ought to be a future strategic priority for planners. In the light of the rapidly urbanizing tendencies, and the severe nature of global challenges that we collectively face (climate change, water scarcity, land degradation to name just a few), better approaches to urban design need to be found than just bricking up the suburbs with mega malls, sports centres and single-use residential zones.

Though respondents are highly aware of their changing environment, particularly the expansion of housing, they are also very much a product of their environment. The suburban expansion of CV has included the development of sports facilities and shopping malls which are an important part of the respondents’ lives. However, alongside their desire for more shops and services, they do also express concern about threats that this transformation brings, e.g., Las Cuevas, a natural area threatened by urban development.

Differences in landscape values attributable to respondents’ age are somewhat inconclusive, though older adolescents did seem to be more aware of landscape values outside of the town than younger ones, something that supports findings from earlier studies and fits with the observation about the emergence of awareness of landscape as a function of maturity.

However, gender-related differences are marked and probably reflect modern society’s concern for the safety of young women, and perhaps also the persistence of conservative societal values that offer male adolescents more opportunities outside of the home than females. With respect to gender equality, especially in the light of growing concerns over health problems resulting from increasingly sedentary lifestyles, the disparity observed in our results, which supports findings from earlier studies, suggests that more needs to be done to encourage girls and young women to participate in physical activities outside the home.

Finally, the study highlights the difficulty presented by PPGIS datasets where points are positioned at multiple scales, and hints at the underlying theoretical weakness of cluster-based analytical approaches in cases where scale cannot be precisely determined.

## Appendix 1: See Table 3

**Table 3:** Land use and land cover changes in the core study area (see Fig. 1), 2005–2011

2011 (Columns)														
2005 (Rows)	Bare land	Crops	Housing	Industrial or extractive	Infrastructures	Other urban	Other urban including housing	Pasture or grassland	Shrubland	Under construction	Waste dumps	Water	Woodland	
Bare land	3768	0	0	0	0	0	0	0	0	0	0	0	0	3768
Crops	0	809	0	0	0	0	0	23	0	0	3	0	0	835
Housing	0	0	1874	0	0	2	1	0	0	0	0	0	0	1877
Industrial or extractive	0	0	0	365	0	0	0	0	3	0	0	0	0	368
Infrastructures	0	0	0	0	254	0	0	0	0	0	0	0	0	254
Other urban	0	0	55	2	0	1678	52	2	0	0	10	0	0	1799
Other urban including housing	0	0	12	0	0	8	610	0	0	0	0	0	0	630
Pasture or Grassland	0	26	4	6	0	219	3	27,862	1	0	206	0	1	28,328
Shrubland	0	0	3	0	0	4	4	3	11,936	0	0	0	1	11951
Under construction	0	0	0	0	0	0	2	0	0	17	0	0	0	19
Waste dumps	0	0	17	5	61	51	20	36	33	0	244	0	0	467
Water	0	0	0	0	0	0	0	0	0	0	0	1324	0	1324
Woodland	0	0	1	0	0	39	1	0	0	0	8	0	11,654	11,703
	3768	835	1966	378	315	2001	693	27,926	11,973	17	471	1324	11,656	63,323

Source: Land Cover Information System for Spain (SIOSE)

## Appendix 2: See Table 4

**Table 4:** List of statistical test and results

No.	Test description	Sample size	Test	Test statistic/parameter	*P* value
1	Association between questions answered and age (all questions)	4343	*X*^2^	62.88 (df = 65)	0.55
2	Association between questions answered and age (activities)	1729	*X*^2^	13.49 (df = 15)	0.56
3	Association between questions answered and age (emotions)	2162	*X*^2^	38.95 (df = 35)	0.30
4	Association between questions answered and age (changes)	2002	*X*^2^	38.14 (df = 55)	0.96
5	Association between questions answered and gender (all questions)	4320	*X*^2^	14.33 (df = 13)	0.35
6	Association between questions answered and gender (activities)	1716	*X*^2^	8.18 (df = 3)	0.04*
7	Association between questions answered and gender (emotions)	2154	*X*^2^	3.08 (df = 7)	0.87
8	Association between questions answered and gender (changes)	2000	*X*^2^	4.31 (df = 11)	0.96
9	Association between specific landscape preferences/change perceptions and land cover	5790	*X*^2^	367.79 (df = 16)	<0.001***
10	Association between respondents’ age and mapped land cover type (activities)	1580	*X*^2^	47.21 (df = 15)	0.001**
11	Association between respondents’ age and mapped land cover type (emotions)	1714	*X*^2^	24.71 (df = 10)	0.006**
12	Association between respondents’ age and mapped land cover type (changes)	1937	*X*^2^	38.82 (df = 25)	0.04*
13	Association between respondents’ gender and mapped land cover type (activities)	1683	*X*^2^	25.56 (df = 8)	0.001**
14	Association between respondents’ gender and mapped land cover type (emotions)	2119	*X*^2^	10.29 (df = 11)	0.50
15	Association between respondents’ gender and mapped land cover type (changes)	2031	*X*^2^	12.13 (df = 8)	0.15
16	Association between changes located by respondents and changes recorded by land cover map (SIOSE)	1240	*X*^2^	97.92 (df = 3)	<0.001***
17	Association between perception of changes relating to expansion and real expansion	804	*X*^2^	98.28 (df = 3)	<0.001***
			Fisher’s Exact	Two-sided	<0.001***
18	Association between perception of changes relating to expansion and real expansion, specified by expansion type	158	*X*^2^	54.02 (df = 6)	<0.001***
			Fisher’s Exact	Two-sided	<0.001***
19	Association between perception of changes relating to abandonment and real abandonment	341	*X*^2^	2.09 (df = 2)	0.35
			Fisher’s Exact	Two-sided	0.38
20	Association between gender and choice of shopping mall or bullfighting arena	46	*X*^2^	0.4 (df = 1)	0.52
21	Association between land cover type and desired future changes	446	*X*^2^	8.39 (df = 2)	0.02*
22	Association between zoom level and landscape preferences/changes mapped	6316	*X*^2^	257.36 (df = 8)	<0.001***

Grey denotes null hypothesis accepted (no significant association detected)

**p* < 0.05; ***p* < 0.01; ****p* < 0.001
